# A Pan‐Cancer Study of Tumour‐Associated Efferocytosis Core Genes and Preliminary Exploration of TIMD4 in Renal Cell Carcinoma

**DOI:** 10.1111/jcmm.70671

**Published:** 2025-06-27

**Authors:** Hongze Li, Jiaqi Zhang, Yixiao Zhang, Zixuan Li, Yuxi Han, Yu Lun

**Affiliations:** ^1^ Department of Urology The First Affiliated Hospital of China Medical University Shenyang China; ^2^ Department of Orthopedics The First Affiliated Hospital of China Medical University Shenyang China; ^3^ Department of Surgical Oncology and General Surgery Key Laboratory of Precision Diagnosis and Treatment of Gastrointestinal Tumors, Ministry of Education, the First Affiliated Hospital of China Medical University Shenyang China; ^4^ Department of Pathology and Pathophysiology College of Basic Medical Sciences, China Medical University Shenyang China; ^5^ Department of Gastroenterology The First Affiliated Hospital of China Medical University Shenyang China; ^6^ Department of Vascular Surgery The First Affiliated Hospital of China Medical University Shenyang China

**Keywords:** efferocytosis, immune microenvironment, immunotherapy, non‐cancer diseases, pan‐cancer, prognosis

## Abstract

Efferocytosis plays a crucial role in maintaining the stability of the immune microenvironment. Increasing research indicates that efferocytosis core genes (ECGs) are not only widely expressed in immune cells, but also expressed at significant levels in tumour cells, impacting tumour progression. However, the comprehensive effects of ECGs on cancer prognosis and the immune microenvironment remain underexplored. This study analysed data from 33 tumour types to assess the expression, diagnostic and prognostic values of ECGs as well as gene modification landscapes. It also predicted the correlation between ECGs, the immune microenvironment and responses to immunotherapy, revealing that lower ECGs expression levels were associated with better immunotherapy responses in various cancers. Additionally, molecular docking studies simulated the interactions between TIMD4 and its sensitive drugs, facilitating targeted drug development. Finally, both in vitro and in vivo experiments confirmed that silencing TIMD4 could effectively inhibit the proliferation and invasion of renal cell carcinoma. Overall, TIMD4 can be used as an important biomarker for tumour prognosis and immunotherapy response, providing new insights into the mechanisms of tumour immune microenvironment and progression, as well as a novel therapeutic target.

**Abbreviations**
ACC: adrenocortical cancer
AD: alzheimer's disease
AF: atrial fibrillation
AMI: acute myocardial infarction
ARTR: acute renal transplant rejection
AUC: area under curve
BLCA: bladder cancer
BRCA: breast cancer
CAVD: calcific aortic valve disease
CESC: cervical cancer
CHOL: bile duct cancer
CKD: chronic kidney disease
COAD: colon cancer
CSU: chronic spontaneous urticaria
CTRP: cancer therapeutics response portal
DFI: disease‐free interval
DLBC: large B‐cell lymphoma
DSS: disease‐specific survival
EAS: efferocytosis associated score
ECGs: efferocytosis core genes
ESCA: oesophageal cancer
FPKM: fragments per kilobase million
GBM: glioblastoma
GDSC: genomics of drug sensitivity in cancer
GSEA: gene set enrichment analysis
HNSC: head and neck cancer
IA: intracranial aneurysm
ICIs: immune checkpoint inhibitors
KICH: kidney chromophobe
KIRC/ccRCC: kidney/renal clear cell carcinoma
KIRP: kidney papillary cell carcinoma
KM: kaplan–meier
LAML: acute myeloid leukaemia
LGG: lower grade glioma
LIHC: liver cancer
LUAD: lung adenocarcinoma
LUSC: lung squamous cell carcinoma
MESO: mesothelioma
Neg.: negative
NOA: non‐obstructive azoospermia
OS: overall survival
OV: ovarian cancer
PAAD: pancreatic cancer
PCA: principal component analysis
Pos.: positive
PRAD: prostate cancer
READ: rectal cancer
RIRI: renal ischemia reperfusion injury
ROC: receiver operating characteristic curve
SARC: sarcoma
SKCM: melanoma
SLE: systemic lupus erythematosus
SONFH: steroid‐induced osteonecrosis of the femoral head
STAD: stomach cancer
TCGA: the cancer genome atlas
THCA: thyroid cancer
TMB: tumour mutational burden
UCEC: endometrioid cancer
UVM: ocular melanomas

## 1 Introduction

Globally, the incidence and mortality rates of cancer continue to rise, making it the second leading cause of death after cardiovascular diseases [1]. As of 2023, it is estimated that 609,820 patients in the United States will die from cancer [2]. The formation of the tumour is not a singular molecular event but rather a complex process involving various factors such as metabolic reprogramming, cell immortalisation and the tumour microenvironment [3]. The complex pathogenesis of cancer has prompted medical scientists to delve deeper into research in hopes of improving treatment outcomes. By utilising public databases such as The Cancer Genome Atlas (TCGA) for pan‐cancer analysis, researchers can uncover clinically valuable biomarkers that can be used for early cancer diagnosis, prognostic assessment and immunotherapy, aiming to significantly enhance patient survival rates. However, during treatment, cytotoxic side effects and issues of drug resistance still have a significant impact on patient prognosis [4]. Despite notable advancements in immunotherapy in recent years, such as the successful application of immune checkpoint inhibitors, the 5‐year overall survival rate for patients has not yet reached an ideal level [5, 6]. Therefore, in the face of cancer as a global public health challenge, there is an urgent need to strengthen the close integration between basic research and clinical applications to promote the development of novel diagnostic and therapeutic strategies, thereby effectively improving the prognosis and survival rates of cancer patients.

The process of efferocytosis refers to the phagocytosis and degradation of apoptotic cells by phagocytes. This process is divided into four steps: first, apoptotic cells release ‘find me’ signals; then, apoptotic cells release ‘eat me’ signals; subsequently, phagocytes internalise and degrade the apoptotic cells; finally, the apoptotic cells are successfully degraded [7]. The homeostasis of the immune microenvironment relies on the clearance of abnormal cells; thus, efferocytosis helps maintain the stability of the immune microenvironment while also creating an immunosuppressive environment that facilitates tumour immune evasion [8]. Additionally, the onset of many non‐tumour diseases is also associated with instability in the immune microenvironment, such as systemic lupus erythematosus (SLE) and sepsis [9, 10]. Therefore, a comprehensive analysis of the role of efferocytosis in diseases can provide meaningful insights for the development of targeted therapeutic strategies in clinical practice.

This study systematically identified eight genes that play pivotal regulatory roles in efferocytosis through a comprehensive review of previous research achievements, designated as Efferocytosis Core Genes (ECGs). Utilising multi‐omics data analysis approaches, we systematically investigated the expression profile characteristics, genetic mutation patterns and transcriptional regulatory mechanisms of ECGs in tumorous versus non‐tumorous diseases compared to normal tissues. Concurrently, by integrating immunohistochemical data, this research profoundly elucidated the differential expression patterns of ECGs and further evaluated their potential impacts on tumour immune microenvironments. Furthermore, the research team comprehensively assessed the clinical applicability of ECGs in prognostic prediction for human cancers and immunotherapy efficacy evaluation through multi‐omics data analysis. During literature investigation, we identified significant research gaps regarding TIMD4's tumour biological functions and pharmacological development. Given TIMD4's characteristic up‐regulation as a prognostic risk factor in renal carcinoma, systematic protein–drug molecular docking studies were conducted alongside functional validation through in vivo and in vitro experiments to explore TIMD4's druggable targets and verify its cancer‐promoting mechanisms. These series of investigations not only provide novel perspectives for understanding ECG biological functions but also establish theoretical foundations for developing therapeutic strategies targeting related diseases.

## 2 Materials and Methods

### 2.1 Download and Processing of Gene Sequencing Data and Clinical Data

The differential analysis of 33 types of cancer and their adjacent samples in this study is based on The Cancer Genome Atlas (TCGA) database (https://portal.gdc.cancer.gov/). The expression data and clinical data for the 33 types of cancer were obtained from the UCSC Xena database (http://xena.ucsc.edu/), where the gene sequencing data have been standardised to fragments per kilobase million (FPKM) format for subsequent analysis. Additionally, the mutation data and methylation modification data in this study were also downloaded from the TCGA database.

We also downloaded gene sequencing data and clinical data for 15 non‐oncology diseases from the Gene Expression Omnibus (GEO) database (https://www.ncbi.nlm.nih.gov/gds/). In addition, we downloaded sequencing data and clinical data for the GSE91061 and GSE93157 cohorts from the GEO database.

### 2.2 Differential Protein Expression Analysis and Immunohistochemical Staining

The results of the differential protein expression analysis are based on the CPTAC database (https://pdc.cancer.gov/pdc/). We downloaded the analysis results from the UALCAN database (https://ualcan.path.uab.edu/) to compare the differential protein expression of ECGs. Additionally, immunohistochemical staining from the HPA database (https://www.proteinatlas.org/) was utilised to compare the expression differences between tumour and normal tissues [11].

### 2.3 Diagnostic and Prognostic Value Assessment of ECGs

We used the R package ‘pROC’ to plot the ROC curves of each gene in tumour and non‐tumour diseases and the area under the curve (AUC) was used to assess the diagnostic value, with an AUC close to 1 indicating a higher diagnostic value.

The UCSC Xena database was used to download overall survival (OS), disease‐specific survival (DSS), progression‐free interval (PFI) and disease‐free interval (DFI) data, and each gene was evaluated for its diagnostic value using univariate Cox regression to assess the effect of each gene on OS, DSS and PFI. The median value was then used as the cut‐off point to categorise each cancer cohort into high‐ and low‐expression groups, and finally, the Kaplan–Meier curve was used to compare the difference in DFI between high‐/low‐expression groups.

### 2.4 Gene Set Enrichment Analysis (GSEA)

The R package ‘clusterProfiler’ was utilised for the GSEA of the core apoptotic gene set. We calculated the enrichment scores for ECGs and divided the samples of each cancer type into high‐ and low‐expression groups based on the median value. Subsequently, we compared the gene expression differences between the two groups and finally performed enrichment analysis of the differential genes based on the HALLMARK gene set.

### 2.5 Construction of Efferocytosis‐Associated Scores (EAS)

The principal component analysis (PCA)‐based scoring methodology was implemented to calculate the efferocytosis‐associated score (EAS). PCA dimensionality reduction was first applied to pan‐cancer efferocytosis core gene expression profiles, utilising linear transformation to project high‐dimensional data into a reduced subspace while maximising variance retention [12]. Ten principal components were extracted, with prioritised focus on the first two components (PC1 and PC2) that cumulatively accounted for the majority of variance. Gene expression values were weighted by PCA loadings to compute principal component scores: ΣPCA_A (PC1 score) = ∑(Gene Expression × PC1 Loading), ΣPCA_B (PC2 score) = ∑(Gene Expression × PC2 Loading). The final EAS was derived through linear combination: EAS = ΣPCA_A + ΣPCA_B. Cohort stratification was performed using median EAS as the classification threshold, partitioning pan‐cancer populations and 15 non‐neoplastic disease cohorts into distinct high‐EAS and low‐EAS subgroups.

### 2.6 Analysis of Immunotherapy Efficacy and the Immune Microenvironment

Using the ‘Estimate’ package, we calculated the ESTIMATE scores, immune scores and stromal scores for each sample and analysed their correlation with EAS. The immune therapy cohorts GSE91061 and GSE93157 were utilised to compare the differences in immunotherapy efficacy between high and low EAS groups. The scores from the TIDE database (http://tide.dfci.harvard.edu/) were used to evaluate the immunotherapy efficacy. Meanwhile, the IPS scores from the TCIA database (https://tcia.at/home) were used to assess the effects of anti‐PD‐1 and anti‐CTLA4 treatments.

The CIBERSORT, EPIC, MCPCOUNTER and QUANTISEQ algorithms were used to analyse the immune cell infiltration of each cancer and their correlation with EAS was also analysed. In addition, the CIBERSORT algorithm was used for the analysis of the immune microenvironment landscape of non‐tumour diseases.

### 2.7 Drug Sensitivity Analysis and Molecular Docking

Gene expression data and corresponding drug sensitivity data for cancer cells were downloaded from the Genomics for Drug Sensitivity in Cancer (GDSC) database (https://www.cancerrxgene.org/) and the Cancer Treatment Response Portal (CTRP) database (http://portals.broadinstitute.org/ctrp/), and correlation analyses were performed based on the GSCA platform (https://guolab.wchscu.cn/). The CellMiner database (https://discover.nci.nih.gov/cellminer/) collects expression data of various drugs acting on 60 types of cancer, and the correlation between TIMD4 gene expression and drug sensitivity was analysed based on the CellMiner database.

The simulation of protein–drug interactions was performed using the 3D structure model data of drugs and proteins in the PubChem database (https://pubchem.ncbi.nlm.nih.gov/) and Protein Data Bank (PDB) database, and based on the CB‐DOCK2 online platform (http://clab.labshare.co.uk/cb‐dock/php/index.php) for molecular docking. CB‐DOCK2 is interconnected with Autodock Vina, the mainstream docking software and also ranks the binding modes according to the Vina score and provides 3D images of the binding modes [13, 14]. The absolute values of binding energies are ranked from highest to lowest.

### 2.8 Cell Culture

We purchased HK2, 769‐P, ACHN and Caki‐1 cell lines from the Shanghai Cell Bank of the Chinese Academy of Sciences for subsequent cell experiments. HK2 cells were cultured in Dulbecco's Modified Eagle Medium (DMEM). Caki‐1 cells were maintained in McCoy's 5A medium. 769‐P cells were grown in RPMI‐1640 medium. ACHN cells were cultured in Minimum Essential Medium (MEM). All the media were mixed with heat‐inactivated foetal bovine serum (FBS) at a ratio of 9:1 and the cells were incubated at 37°C under 5% CO_2_.

### 2.9 Constructing Stable Cell Lines With shRNA

In order to generate TIMD4 stable knockdown cell lines, 769‐P and ACHN cells were inoculated into six‐well plates, respectively, and when the fusion level reached about 60%, shRNA targeting TIMD4 was transfected into renal carcinoma cell lines using a reverse transcription virus and finally screened using puromycin for 2 weeks. Finally, the stable cell lines were continued to be cultured and passaged for protein extraction to verify the knockdown effect and subsequent functional experiments. shTIMD4 sequence was:

shTIMD4#1(Sense): 5’‐CCCATGTCAATGAAGAATGAA‐3’

shTIMD4#2(Sense): 5’‐GATGTCTCCTTGACCATCTTA‐3’

### 2.10 Knockdown of TIMD4 Using siRNA

Gene silencing was performed using human renal carcinoma cell lines 769‐P and ACHN. Cells in logarithmic growth phase were plated in six‐well plates at optimised densities (769‐P: 4 × 10^4^ cells/well; ACHN: 6 × 10^4^ cells/well) and transfected at 60%–70% confluence. siRNA (20 nM) and Lipo3000 transfection reagent were separately diluted in serum‐free RPMI‐1640 (for 769‐P) and MEM (for ACHN) media at a 1:1 volume ratio, incubated at room temperature for 5 min, then combined and allowed to form complexes for 15 min. Following medium removal, 1.5 mL complex‐containing medium was added per well, with subsequent replacement by complete medium after 6‐h incubation at 37°C. Cells were harvested at 24 h (769‐P) or 48 h (ACHN) post‐transfection for mRNA expression analysis by quantitative reverse transcription PCR (qRT‐PCR). The siRNA sequence targeting TIMD4 was:

siTIMD4(Sense): 5’‐GUUCAACGAUGUAAAGAUAAA −3’

siTIMD4(Antisense): 5’‐UAUCUUUACAUCGUUGAACCA −3’

### 2.11 Western Blot of the Target Protein

Cancer cells were lysed using RIPA lysis buffer to extract total proteins and the proteins were quantified. Equal amounts of protein samples were separated by SDS‐PAGE and transferred to PVDF membranes. The membrane was closed in 3% bovine serum albumin at 37°C for 1 h. The membrane was then incubated with primary antibody overnight at 4°C and finally with secondary antibody at 37°C for 40 min and the experiment was repeated 3 times.

### 2.12 RT‐qPCR

Total RNA from the above cells was extracted using TRIzol reagent (Invitrogen). cDNA was obtained by reverse transcription using PrimeScript RT premix (Takara). β‐Actin was used as an internal reference for RT‐qPCR and the sequence of the TIMD4 primer was as follows:

Forward: 5’‐GTACTGCTGCCGCATAGAAGT‐3’

Reverse: 5’‐TTGTTGTCATTTGTCGGGTGG‐3’

### 2.13 Wound Healing and Transwell Assays

Wound healing and Transwell assays were used to assess the migratory capacity of cells. For the wound healing assay, a marker was used to draw guideline lines on the bottom of a six‐well plate. Renal cancer cells were digested with trypsin, re‐suspended and seeded into the six‐well plate. Once the cells covered the bottom of the plate, a ruler was used to plan the scratch path and a 10‐μL pipette tip was used to create scratches along the planned path, with the scratches oriented perpendicular to the guideline lines. The cells were then washed three times with PBS to remove suspended cells and photographs were taken at 0 and 48 h post‐scratch. The cells were cultured in serum‐free medium and the area of wound healing was calculated using Image J software. This assay was repeated 3 times.

For the Transwell assay, to eliminate the influence of serum, renal cancer cells in the NC group and sh group were first starved in serum‐free medium for 4 h, then digested with trypsin and re‐suspended in serum‐free medium. The cell suspension was added to the upper chamber of the Transwell insert, while the lower 24‐well plate was filled with complete medium containing 10% FBS. After incubating at 37°C for 48 h, the cells were washed three times with PBS, fixed with 4% paraformaldehyde for 30 min and then stained with crystal violet for 30 min. Finally, the cells were washed three times with PBS and non‐invading cells in the upper chamber were removed with a cotton swab. Photographs were taken under a microscope at 200 × magnification and the experiment was repeated 3 times. Cell counting was performed using Image J software.

### 2.14 CCK‐8 and Colony Formation Assays

CCK‐8 and colony formation assays were used to assess cell proliferation capacity. For the CCK‐8 assay, tumour cells were digested and re‐suspended, followed by counting using a cell counting plate 3 times to obtain an average value. Then, sh‐TIMD4‐transfected cells and control group cells were seeded into a 96‐well plate at a density of 2000 cells per well. The experiment was divided into five time points: 0, 24, 48, 72 and 96 h, with six replicates for each time point. At each time point, 10 μL of CCK‐8 solution was added to each well and the solution was mixed gently using a pipette. The 96‐well plate was then wrapped in aluminium foil and incubated in the dark at 37°C for 4 h. Finally, the absorbance at 450 nm was measured using a multimode plate reader and recorded.

For the colony formation assay, the counted sh‐TIMD4 and control group cells were seeded at a density of 1000 cells per well in a six‐well plate and cultured in complete medium at 37°C in a 5% CO_2_ environment for 7 days. Afterward, 4% paraformaldehyde was added for fixation for 30 min, followed by staining with crystal violet for 30 min. The wells were then washed 3 times with PBS and allowed to dry. Finally, photographs of each well were taken and colonies were counted using Image J software. Each experiment was repeated 3 times.

### 2.15 Animal Models of Tumour

Six‐week‐old male BALB/C nude mice were purchased from the Animal Experiment Center of China Medical University (Shenyang, China). All animal experiments were approved by the Animal Ethics Committee of China Medical University in accordance with current animal ethics and welfare guidelines. For mouse‐loaded tumour experiments, approximately 5 million ACHN cells were injected subcutaneously into the axilla 5 mm below the armpit of nude mice, with five mice in each group, which were divided into NC and sh groups. Tumour volume was measured every 4 days with the formula V = 0.52*length*width^2^. The endpoint of the experiment was set at 28 days after inoculation or natural death of the mice. At the end of the experiment, mice were executed and tumours were dissected out for subsequent studies.

### 2.16 Data Processing and Image Visualisation

Appropriate statistical methods were selected for data analysis. The *t* test and Wilcoxon rank sum test were used for comparison between groups. Survival curves plotted by Kaplan–Meier method were used to compare the difference in survival rates between the two groups. Spearman's test was used to assess the correlation. Cox analysis was used to calculate the risk ratio (HR) and 95% confidence interval (95% CI). The *p* < 0.05 was considered statistically significant (*p < 0.05, **p < 0.01, ***p < 0.001). Statistical analysis was performed in R software and Graphpad software. Images were visualised using Adobe Illustrator and Image J software.

## 3 Results

### 3.1 Genomic Landscape of ECGs in Pan‐Cancer

**FIGURE 1:**
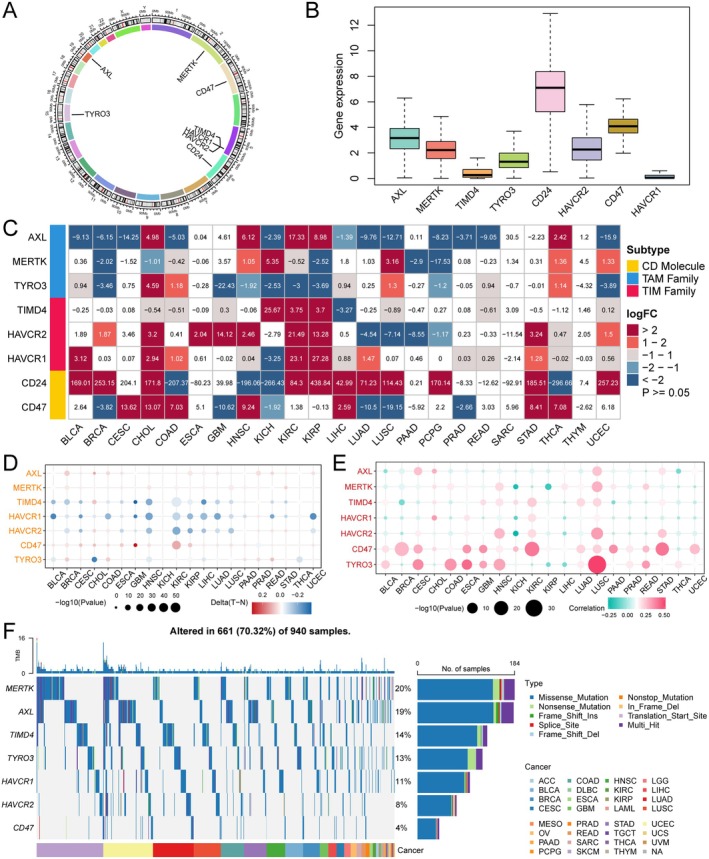
Transcriptomic landscape analysis of ECGs. (A) The positions of eight ECGs on the chromosomes. (B) The expression of ECGs in pan‐cancer samples. (C) Differential expression analysis of ECGs in pan‐cancer samples. (D) Methylation status of ECGs in pan‐cancer samples. (E) Somatic copy number alterations of efferocytosis core genes. (F) Single nucleotide variation status of efferocytosis core genes.

The positions of ECGs on the chromosomes are shown in Figure 1A. Subsequently, we analysed the expression levels of these genes across 33 types of cancer and found that AXL, MERTK, TYRO3, CD24, HAVCR2 and CD47 exhibited higher expression levels, while TIMD4 and HAVCR1 showed relatively lower expression (Figure 1B). We further analysed the expression of ECGs' mRNA in tumour versus normal tissues based on the TCGA database. We observed that the expression of ECGs varied in most tumours, particularly showing significant differences in CHOL, HNSC, KIRC, KIRP, STAD and THCA, whereas no significant differences were found in SARC and THYM compared to normal tissues (Figure 1C). To investigate whether the expression of ECGs is regulated by methylation, we analysed the differences in DNA methylation of these genes in tumour versus normal tissues using TCGA methylation data. We observed that they exhibited complex methylation patterns across 20 cancer types, with only TIM family members consistently showing low methylation in 12 tumours (Figure 1D). We also further analysed the somatic copy number alterations (SCNA) of the eight genes to explore the genetic aberrations of ECGs in cancer. Ultimately, we found that ECGs presented different profiles across 20 cancer types, with CD47 and TYRO3 more frequently exhibiting copy number increases rather than losses in the majority of tumours, while AXL and HAVCR1 showed a relatively opposite trend (Figure 1E). Next, we analysed the single nucleotide variations (SNVs) of ECGs in 31 types of cancer and found that mutations in ECGs occurred in 70.32% of the samples, predominantly as missense mutations. MERTK had the highest mutation rate at 20%, while CD47 had a mutation rate of only 4% (Figure 1F). Overall, ECGs exhibit complex expression and aberration patterns in cancer, playing a significant role in tumours.

### 3.2 Protein Expression of ECGs in Normal and Tumour Tissues

**FIGURE 2:**
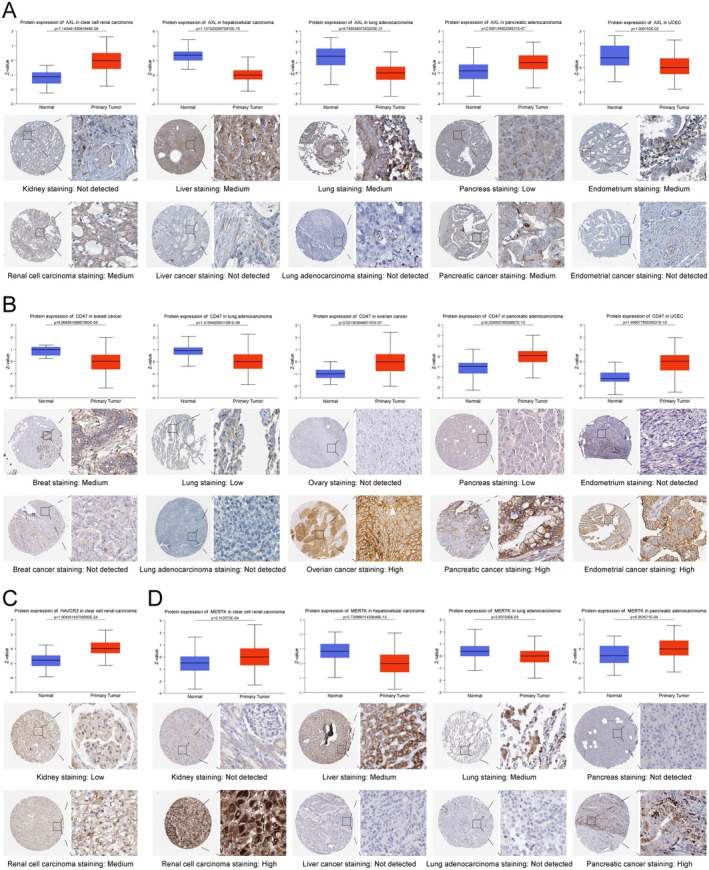
Differences in protein expression of ECGs and immunohistochemical staining. (A) Differences in protein expression of AXL. (B) Differences in protein expression of CD47. (C) Differences in protein expression of HAVCR2. (D) Differences in protein expression of MERTK.

To understand the protein expression levels of ECGs, we analysed the differences in protein expression of ECGs between normal tissues and tumour tissues based on the CPTAC and HPA databases. We found that AXL and MERTK were expressed at higher levels in liver tissue, lung tissue, endometrial tissue (Figure 2A,D); CD47 was up‐regulated in breast tissue, lung tissue, ovarian cancer, pancreatic cancer and endometrial cancer (Figure 2B); and HAVCR2 showed higher expression in renal clear cell carcinoma (Figure 2C). However, AXL and MERTK were up‐regulated in renal clear cell carcinoma and pancreatic cancer (Figure 2A,D). In the meantime, we validated these conclusions using immunohistochemical staining data from the HPA database. From the perspective of protein expression characteristics, ECGs hold significant research value in most tumours.

### 3.3 Assessment of the Diagnostic Value of ECGs in Tumour and Non‐Tumour Diseases

**FIGURE 3:**
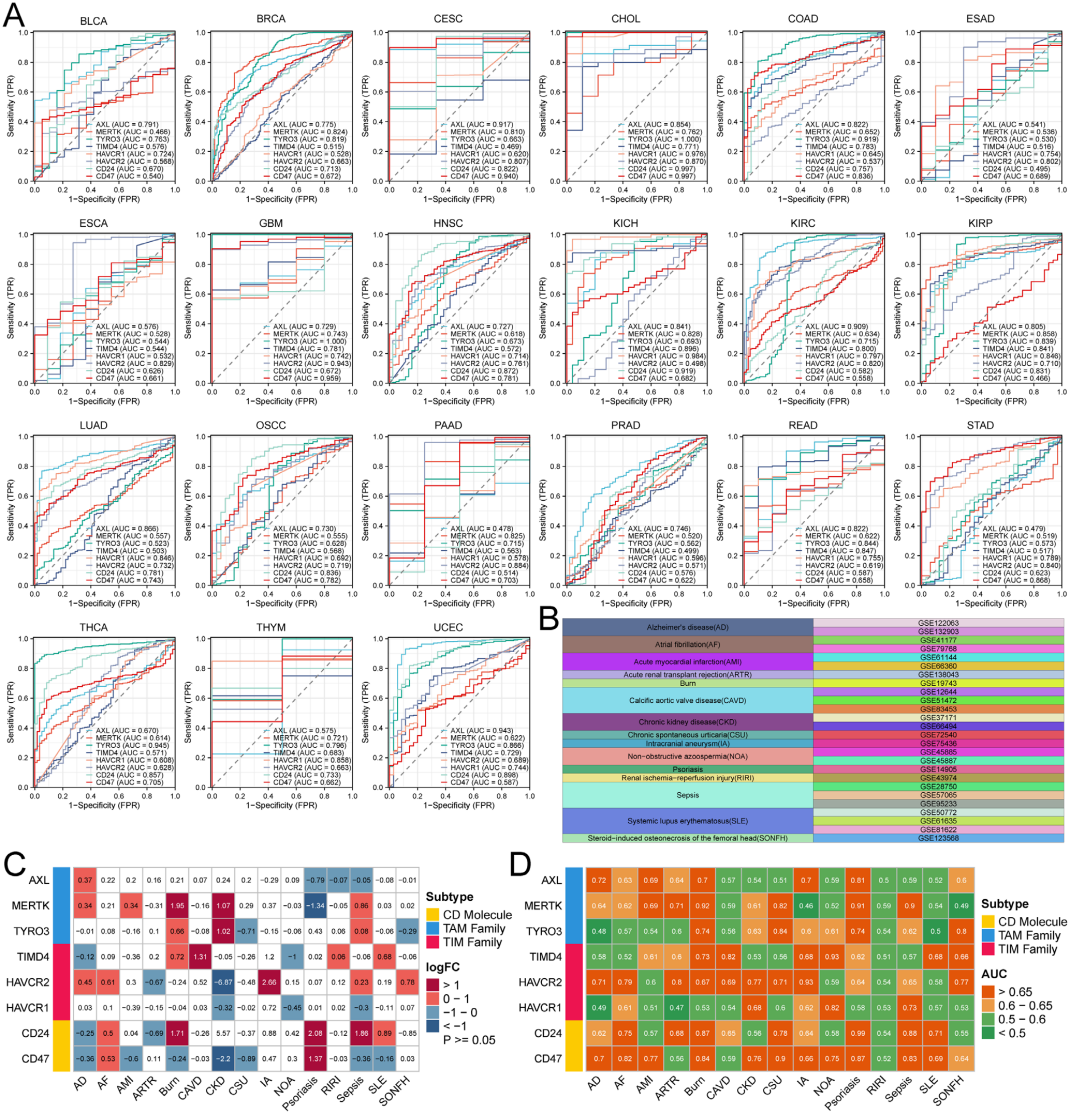
Comparison of the diagnostic value of ECGs in tumour and non‐tumour diseases. (A) ROC curve analysis of the diagnostic value of abnormally expressed ECGs based on TCGA data. AUC < 0.5 is considered to have no diagnostic value, 0.5 ≤ AUC < 0.7 is considered to have low diagnostic value and AUC ≥ 0.7 is considered to have high diagnostic value. (B) Summary of 15 non‐tumour diseases based on the GEO database. (C) Differential expression analysis of ECGs in non‐tumour diseases. (D) ROC analysis of the diagnostic value of abnormally expressed ECGs for different non‐tumour diseases.

We plotted ROC curves to evaluate the diagnostic value of ECGs in tumours, where an area under the curve (AUC) closer to 1 indicates a higher diagnostic value of the gene, suggesting its potential as a diagnostic biomarker. For instance, AXL had AUC values greater than 0.9 in CESC, KIRC and UCEC and even TYRO3 reached an AUC of 1 in GBM. This suggests that members of ECGs can be utilised for cancer diagnosis (Figure 3A). Furthermore, we investigated the diagnostic value of ECGs in 15 non‐tumour diseases based on the GEO database (Figure 3B). First, we analysed the expression of ECGs between disease groups and normal groups, revealing that ECGs exhibited complex expression patterns across the 15 non‐tumour diseases. However, in calcific aortic valve disease (CAVD), only TIMD4 was up‐regulated, while in intracranial aneurysm (IA), only HAVCR2 showed increased expression (Figure 3C). Next, we calculated the AUC values of ECGs in the 15 non‐tumour diseases, finding that ECGs had the highest diagnostic value for burns, with CD24 achieving an AUC of 0.99 in the diagnosis of psoriasis (Figure 3D). These results indicate that ECGs are not only applicable for the diagnosis of tumour diseases, but can also serve as diagnostic biomarkers for non‐tumour diseases.

### 3.4 Evaluation of Prognostic Value of ECGs in Tumour and GSEA

**FIGURE 4:**
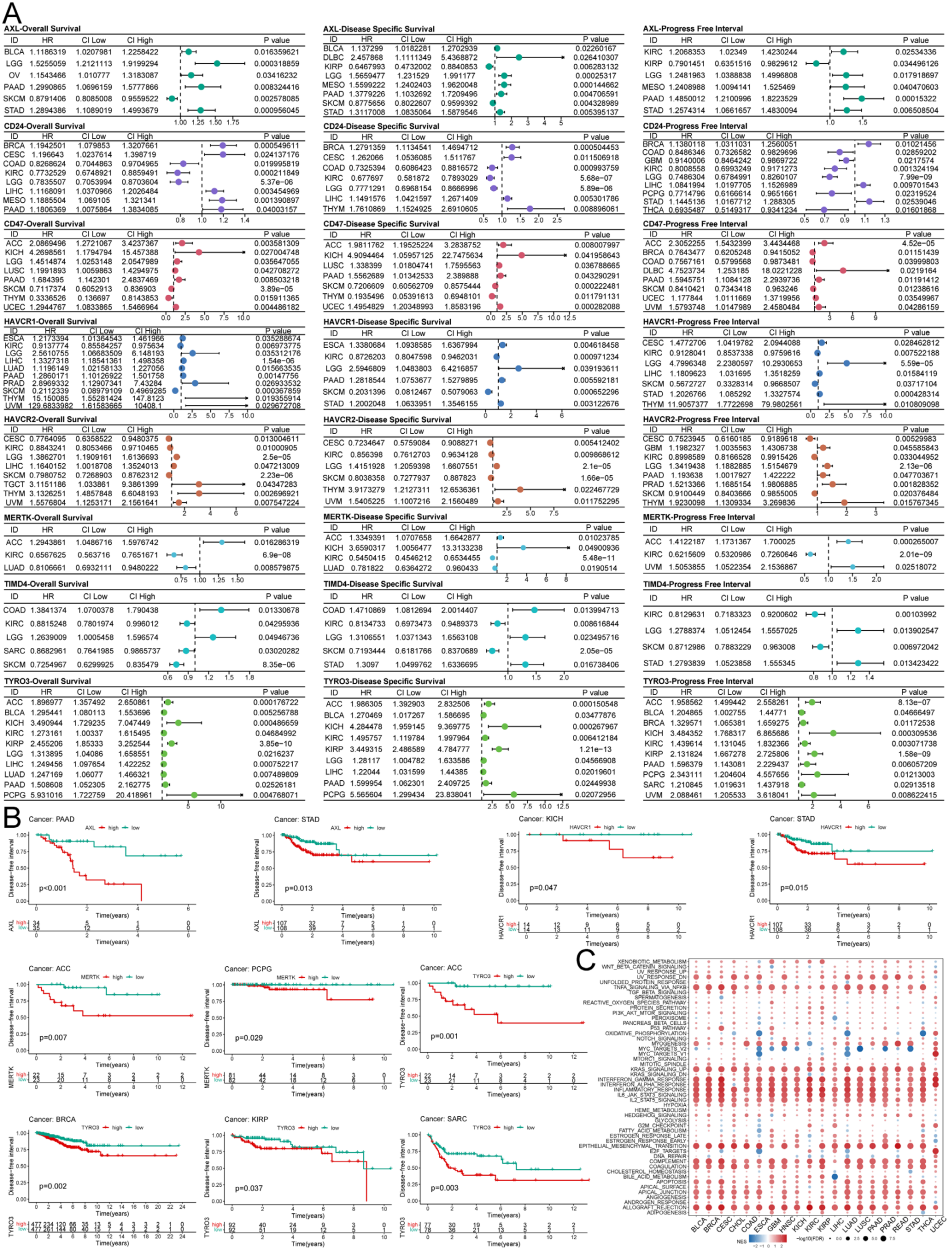
Prognostic analysis and GSEA of ECGs. (A) Univariate Cox analysis of pan‐cancer based on TCGA database. (B) The impact of ECGs on disease‐free interval. (C) GSEA of ECGs based on TCGA pan‐cancer data.

Based on the TCGA database, Unicox regression was used to assess the prognostic value of ECGs in different tumours. The results indicated that most ECGs acted as risk factors associated with the prognosis of LGG and PAAD in terms of overall survival. Additionally, we found that ECGs negatively impacted disease‐specific survival and progression‐free survival in several cancers (Figure 4A). Kaplan–Meier curves were utilised to evaluate the effect of ECGs expression on disease‐free intervals, revealing that high expression of AXL was associated with poor prognosis in PAAD and STAD; HAVCR1 also contributed to worse prognosis in KICH and STAD; both MERTK and TYRO3 were linked to adverse prognosis in ACC (Figure 4B). Overall, ECGs have a detrimental effect on the prognosis of various cancers and can serve as important biomarkers for cancer diagnosis and prognostic evaluation. Finally, to clarify the detailed mechanisms of ECGs in tumour progression, we conducted GSEA. We found that ECGs were significantly enriched in the TNF‐α/NF‐κB, KRAS and IL‐6/JAK/STAT3 pathways across nearly all cancer types. Furthermore, utilising TIMD4‐silenced cell lines, we confirmed the activation of the NF‐κB and STAT3 pathways and demonstrated that TIMD4 silencing markedly inhibited the activation of both pathways (Figure S1A,B). Moreover, it was evident that ECGs are involved in the regulation of epithelial–mesenchymal transition in cancer, which corroborates the notion that ECGs lead to poor tumour prognosis (Figure 4C). In summary, ECGs act as oncogenic factors and their up‐regulation results in poor prognosis for cancer patients, with enrichment observed across various cancer‐related signalling pathways.

### 3.5 Analysis of the Correlation Between Efferocytosis Core Genes and Immunotherapy Efficacy

**FIGURE 5:**
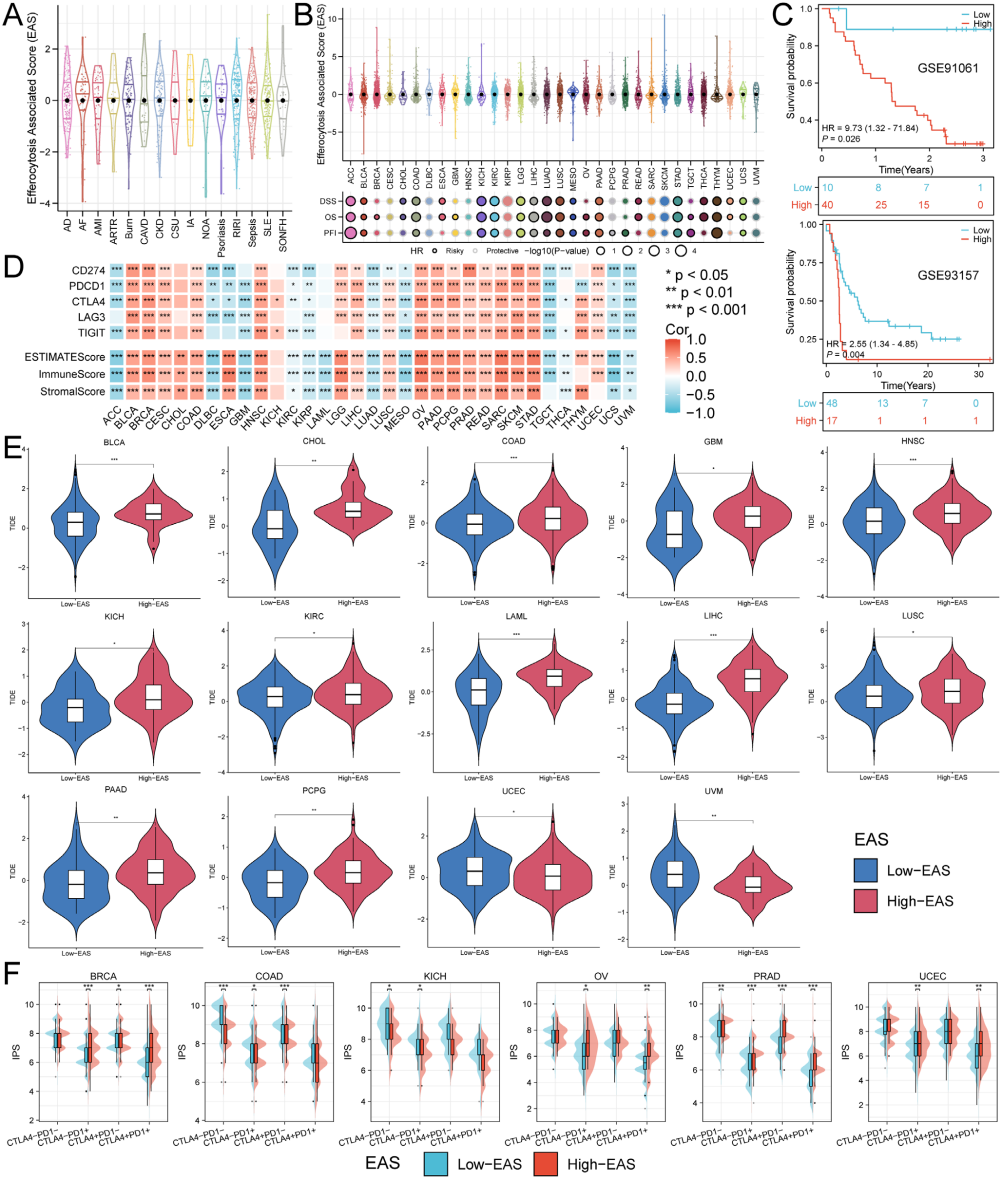
Construction of the EAS and analysis of the correlation with immunotherapy. (A) Construction of EAS in non‐tumour diseases. (B) Construction of EAS and prognostic analysis of EAS based on TCGA pan‐cancer data. (C) Overall survival analysis of EAS in the immunotherapy cohorts GSE91061 and GSE93157. (D) Correlation analysis of EAS with common immune checkpoints and immune‐related scores. (E) Comparison of TIDE scores between high and low EAS groups. (F) Comparison of IPS scores between high and low EAS groups.

To comprehensively assess the expression characteristics of ECGs in each patient, we utilised the PCA algorithm to construct an efferocytosis‐associated score (EAS) (Figure 5A,B). Additionally, we found that EAS had a detrimental impact on the prognosis of many cancers, including renal cancer, colorectal cancer, liver cancer and gastric cancer (Figure 5B). Subsequently, we calculated the optimal cut‐off value to divide patients into high and low EAS subgroups. The GSE91061 cohort documented the effects of anti‐PD‐1 and anti‐CTLA4 immunotherapy in patients with melanoma and non‐small cell lung cancer, while GSE93157 studied the responses of melanoma and lung cancer patients to anti‐PD‐1 treatment. By comparing the survival differences between high and low EAS groups in patients receiving immunotherapy from these two cohorts, we found that the low EAS group was more suitable for immunotherapy (Figure 5C). Through the analysis of the correlation between EAS and immune checkpoint expression, we discovered that EAS was positively correlated with the expression of immune checkpoints in the majority of tumours, with only a small number, such as ACC, DLBC and GBM, showing a negative correlation. In tumour immunotherapy, the tumour microenvironment plays a crucial role in treatment response. We calculated the correlation between EAS and ESTIMATEScore, ImmuneScore and StromalScore, finding that EAS was positively correlated with all three in most tumours. However, in ACC, DLBC, GBM, KIRC, KIRP, LAML, LUAD, MESO, TGCT, THCA, UCS and UVM, EAS showed a negative correlation with these scores (Figure 5D), which also validated the analysis results of the aforementioned immunotherapy cohorts. Next, we also assessed the correlation between EAS and immunotherapy efficacy across various cancers using the TIDE score, revealing that in UCEC and UVM, the low EAS group had better immunotherapy outcomes. Conversely, in BLCA, CHOL, COAD, GBM, HNSC, KICH, KIRC, LAML, LIHC, LUSC, PAAD and PCPG, the high EAS group was more suitable for immunotherapy (Figure 5E). Finally, by comparing the IPS of different EAS populations, we found that in BRCA, OV, PRAD and UCEC, the high EAS group was more suitable for anti‐PD‐1 treatment (Figure 5F). In summary, EAS can predict the immune microenvironment and immunotherapy outcomes, indicating a correlation between ECGs and tumour immunity.

### 3.6 Analysis of the Correlation Between EAS and Immune Cell Infiltration

**FIGURE 6:**
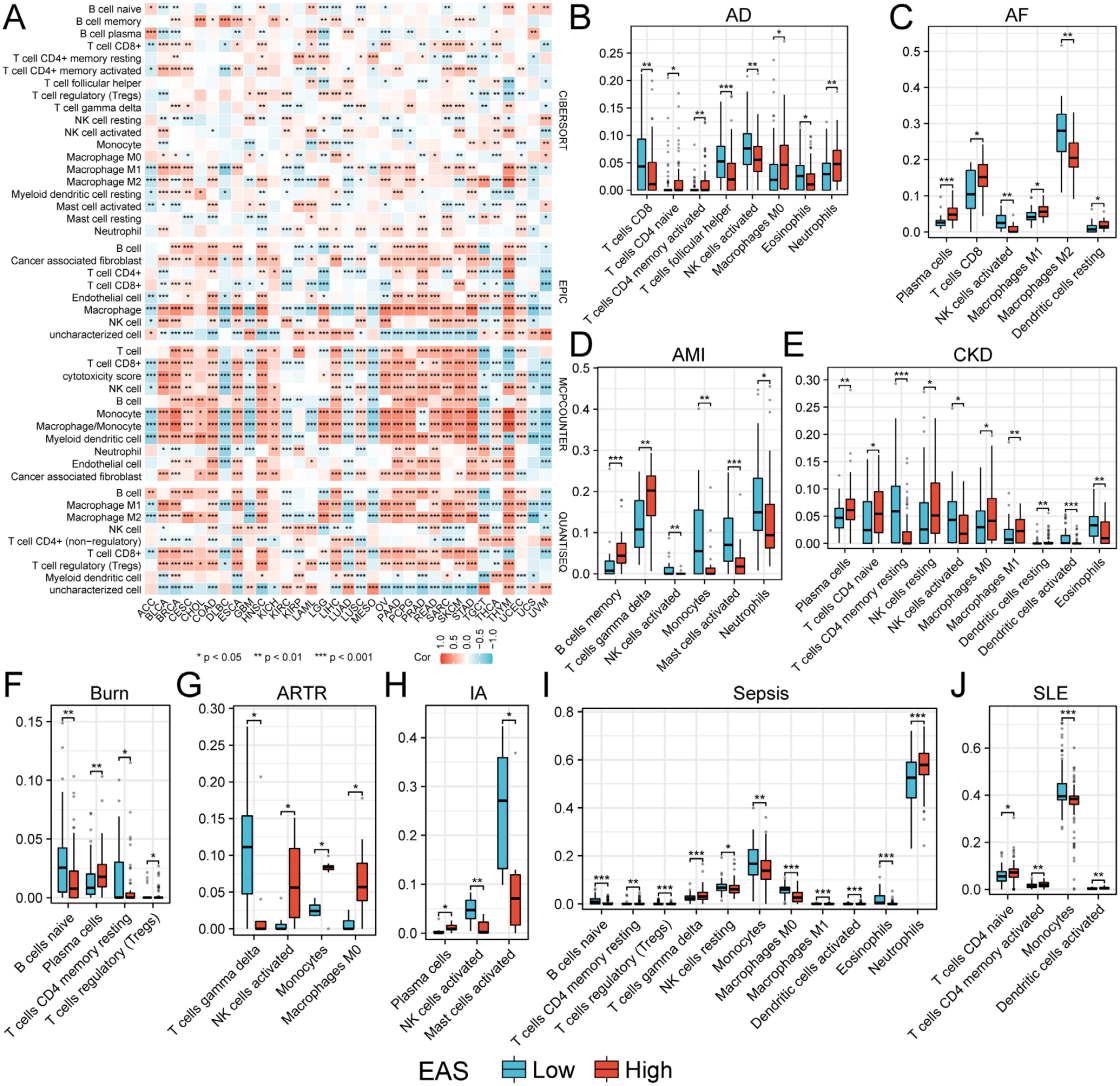
Correlation of the EAS with immune microenvironment. (A) Correlation of immune cell infiltration with EAS in pan‐cancer calculated by different algorithms. (B–J) Differences in immune cell infiltration between high and low EAS groups in non‐tumour diseases.

First, we utilised the CIBERSORT, EPIC, MCPCOUNTER and QUANTISEQ algorithms to analyse the abundance of immune cells in various tumours. We found that in cancers such as BLCA, CHOL, COAD, HNSC and LIHC, the infiltration abundance of immune cells was positively correlated with EAS. Conversely, in LUAD and MESO, most immune cells showed a negative correlation with EAS, which corroborated the previous results (Figure 6A). Given the significant role of immune cells in various non‐tumour diseases, we also compared the abundance of immune cells in non‐tumour diseases (Figure 6B–J). We discovered that EAS was also correlated with the abundance of immune cells in non‐tumour diseases, indicating that EAS has a good predictive effect on the immune microenvironment.

### 3.7 Drug Screening Targeting ECGs

**FIGURE 7:**
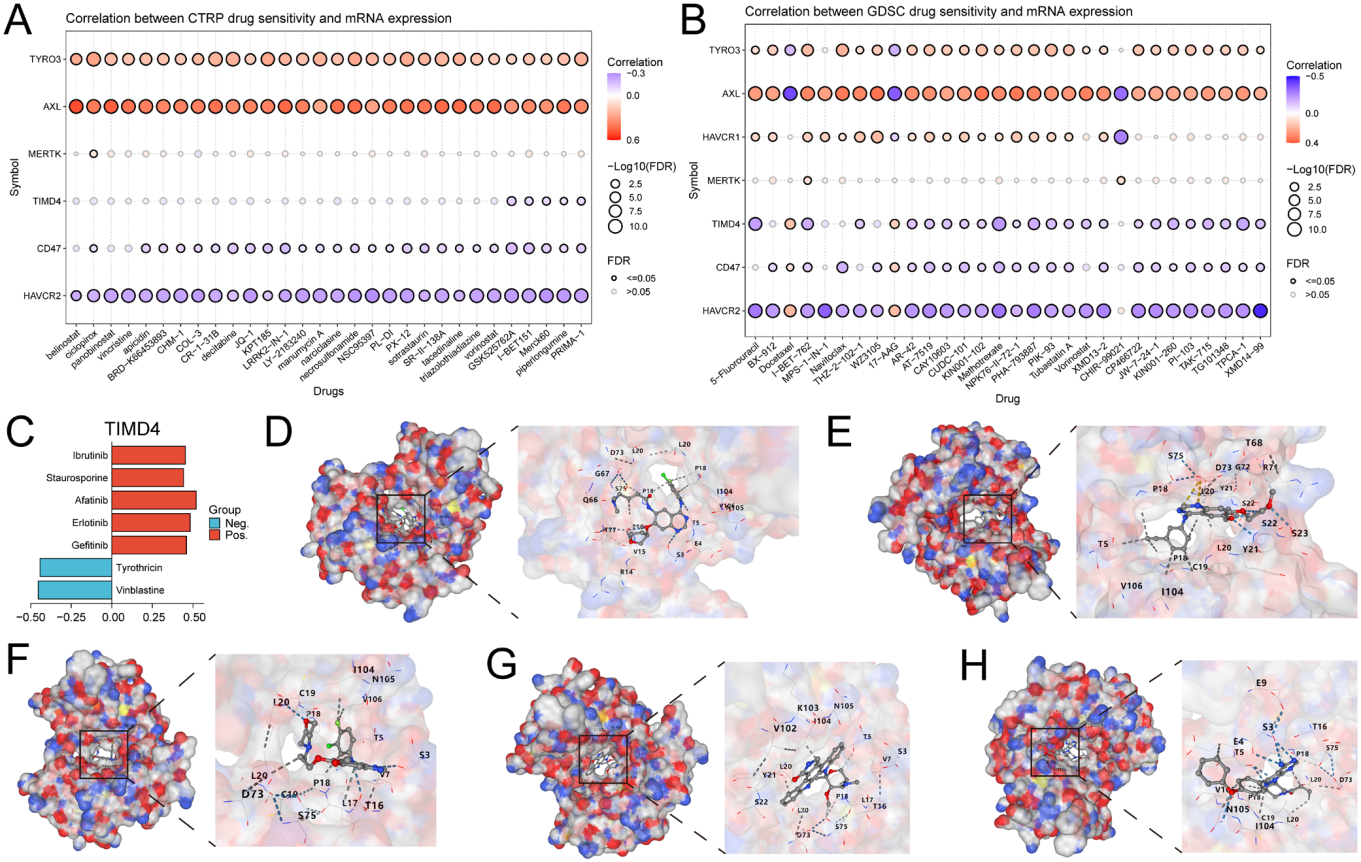
Drug sensitivity analysis and molecular docking of ECGs. (A) Analysis of the correlation between the expression of ECGs and drug sensitivity based on the CTRP database. (B) Analysis of the correlation between the expression of ECGs and drug sensitivity based on the GDSC database. (C) Analysis of the correlation between TIMD4 expression and drug sensitivity based on the CellMiner database. Molecular docking performed using the CB‐DOCK2 online docking platform: (D) afatinib, (E) erlotinib, (F) gefitinib, (G) staurosporine and (H) ibrutinib.

We analysed the correlation between drug sensitivity and the mRNA expression of ECGs based on the CTRP and GDSC databases. The results indicated that sensitivity was positively correlated with the expression of AXL and TYRO3 for the most drugs (Figure 7A,B). Based on currently published studies, we found that the functions and cancer regulatory mechanisms of seven ECGs, excluding TIMD4, have been elucidated. We also compiled a list of drugs that have entered pre‐clinical and clinical trials, discovering that no effective targeted small molecule drugs for TIMD4 have been identified thus far. Therefore, we conducted a drug exploration for TIMD4 based on the Cellminer database (Figure 7C) and identified five small molecules whose sensitivity was positively correlated with TIMD4 expression (afatinib, erlotinib, gefitinib, staurosporine and ibrutinib). Notably, four of these drugs are tyrosine kinase inhibitors. Finally, we performed molecular docking using the CB‐DOCK2 online docking platform, where a lower binding free energy indicates a more stable binding. Consequently, we selected the model with the lowest binding free energy for visualisation (Figure 7D–H).

### 3.8 Validation of TIMD4 Expression and Functional Exploration in Renal Cell Carcinoma

**FIGURE 8:**
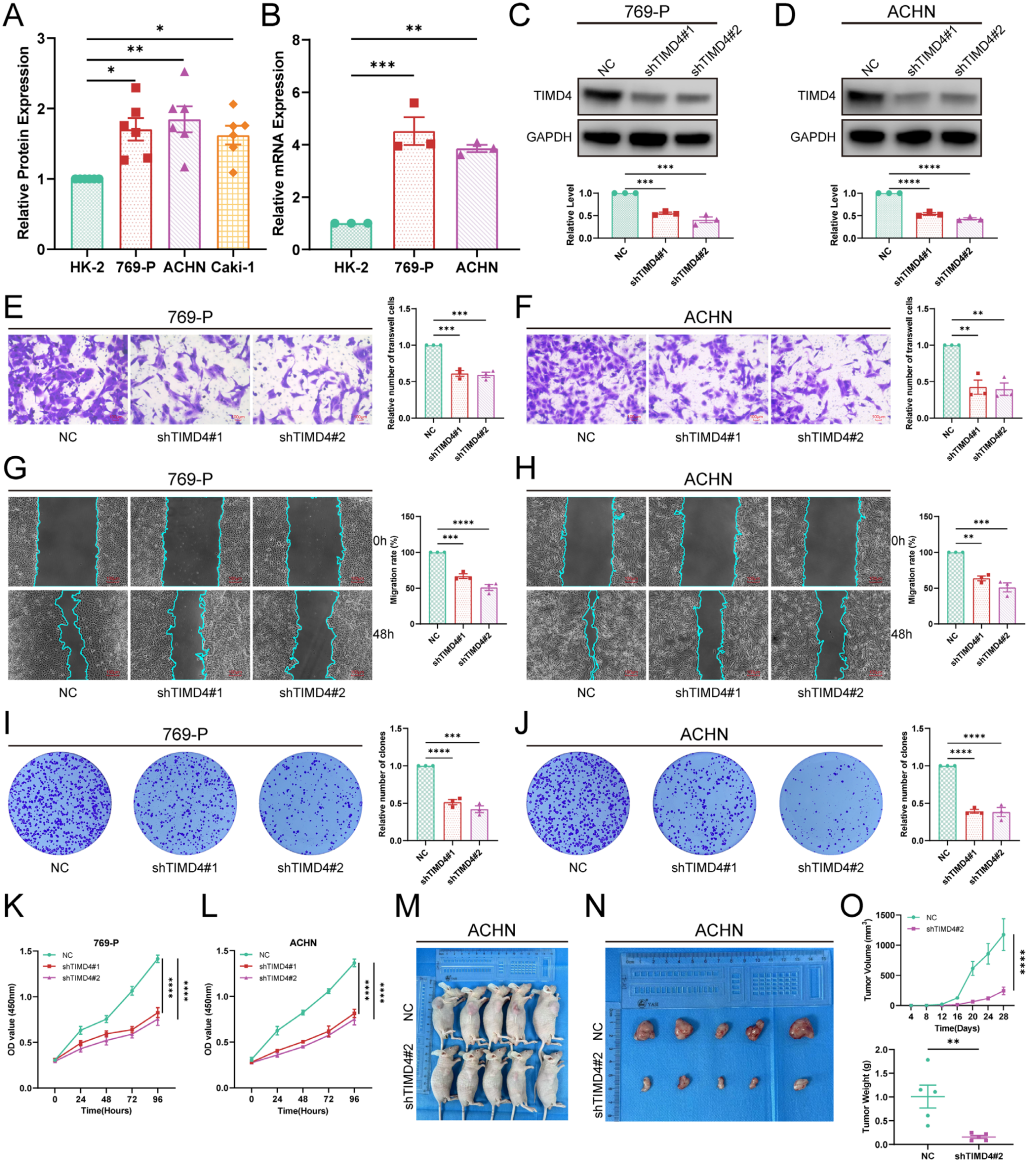
In vitro and in vivo experimental validation of the biological function of TIMD4 in tumour cells. (A) Western blot validation of the protein expression differences of TIMD4 in normal renal cells and renal tumour cells. (B) RT‐qPCR validation of the mRNA expression differences of TIMD4 in normal renal cells and renal tumour cells. (C, D) Verification of the effect of shRNA knockdown of TIMD4 on protein expression in two cell lines. Transwell migration assays (E, F), wound healing assays (G, H), colony formation assays (I, J) and CCK8 assays (K, L) in two stable shTIMD4‐transfected cell lines, 769‐P and ACHN. (M) Photographs of mice in shTIMD4#2 and NC groups. (N) Photographs of tumours dissected from tumour‐bearing mice in the shTIMD4 group and NC group. (O) Tumour volume and tumour weight were used to compare tumour size. *Note*: *p < 0.05, **p < 0.01, ***p < 0.001, ****p < 0.0001.

We extracted total proteins from normal renal cell lines and renal cancer cell lines and validated the protein expression levels using western blot. We found that TIMD4 was expressed at higher levels in 769‐P, ACHN and Caki‐1 cells (Figure 8A), and most significantly in 769‐P and ACHN. To verify the expression of TIMD4 at the RNA level, we extracted total RNA from HK‐2, 769‐P and ACHN cells for RT‐qPCR analysis. The results indicated that TIMD4 expression was up‐regulated in 769‐P and ACHN cells (Figure 8B), leading us to select 769‐P and ACHN for subsequent functional experiments. We further investigated the impact of TIMD4 on the biological behaviour of cancer cells. We used shRNAs (shTIMD4#1, shTIMD4#2) to knock down TIMD4 and the knockdown efficiency was validated by western blot, showing that the shRNA effectively reduced TIMD4 expression (Figure 8C,D). To validate the specificity of TMID4 knockdown, we subsequently performed siRNA‐mediated silencing of TMID4 expression (Figure S1C,D). We then employed Transwell and wound healing assays to assess the effect of TIMD4 on the migratory capacity of tumour cells. Ultimately, we found that TIMD4 knockdown significantly decreased the migratory ability of the cells, indicating that TIMD4 promotes renal cell carcinoma invasion and metastasis by enhancing tumour cell migration (Figure 8E–H). Colony formation assay and CCK8 assay were used to evaluate the proliferative capacity of tumour cells. In the colony formation assay, we observed that the number of cell colonies in the control group was greater than that in the shTIMD4 group, with a more significant difference noted in the ACHN cell line (Figure 8I,J). The results of the CCK‐8 assay also indicated that the knockdown of TIMD4 inhibited the proliferation of tumour cells, with a more pronounced inhibitory effect on the proliferation of the ACHN cell line (Figure 8K,L). Taken together, these findings suggest that TIMD4 promotes the proliferation and migration of renal cell carcinoma cells, with a particularly significant effect on the ACHN cells, which are classified as renal papillary cell carcinoma.

### 3.9 TIMD4 Promotes Tumour Growth In Vivo

Through previous studies, we found that TIMD4 is highly expressed in ACHN and has significant functions. To investigate the impact of TIMD4 on the growth of renal cell carcinoma in vivo, we conducted animal experiments. First, we injected ACHN cells stably transfected with shTIMD4#1 into male BALB/C nude mice and measured the tumour size every 4 days. The results showed that the tumour size in the TIMD4 knockdown group was significantly smaller than that in the NC group (Figure 8M–O), indicating that TIMD4 can also regulate the proliferation of ACHN cells in vivo.

## 4 Discussion

Cancer has become a significant threat to human life [15, 16]. Utilising the biological processes of tumour occurrence and development for targeted screening, diagnosis, treatment and prognostic prediction of tumours is feasible. In recent years, research on apoptosis has increased and three types of ECGs that are significantly expressed in normal and tumour cells have been shown to have substantial clinical value [17, 18]. We compiled three types of ECGs from previous literature and systematically analysed their abnormal expression, gene mutations, methylation regulation, diagnostic and prognostic roles, enrichment status in cancer‐related signalling pathways and correlations with the immune microenvironment and immunotherapy efficacy based on pan‐cancer multi‐omics data [8, 19]. Additionally, we conducted small molecule drug screening for the TIMD4 gene, which has been rarely studied in tumour cells. Our research indicates that the expression of ECGs differs in many tumours and non‐tumour diseases. In terms of gene expression, ECGs showed significant differences in CHOL, HNSC, KIRC, KIRP, STAD and THCA, with notable up‐regulation of ECGs in non‐tumour diseases such as burns and sepsis. Methylation regulation may be a reason for the differences in gene expression in cancer [20]. In terms of protein expression, we found that AXL was significantly up‐regulated in renal clear cell carcinoma, CD47 was significantly up‐regulated in ovarian cancer, pancreatic cancer and endometrial cancer, and MERTK was significantly up‐regulated in both ccRCC and pancreatic cancer. For gene mutations, MERTK had the highest mutation rate, while CD47 had the lowest. Moreover, ECGs play a significant role in the diagnosis of tumours and non‐tumour diseases, as well as in the prognostic prediction of cancer patients, making them viable biomarkers for disease diagnosis and prognosis. Through GSEA of ECGs, we revealed that ECGs exhibit significant enrichment in TNF‐α/NF‐κB, KRAS and IL‐6/JAK/STAT3 signalling pathways across most malignancies, demonstrating strong correlations with the epithelial–mesenchymal transition (EMT) process. The mechanisms potentially involve TNF‐α/NF‐κB‐mediated immune microenvironment remodelling, KRAS‐driven MAPK/ERK pro‐proliferative cascades and sustained IL‐6/JAK/STAT3 pathway activation synergistically inducing key EMT‐related transcription factors, while potentially augmenting tumour cell migratory capacity, invasive potential and distant metastasis predisposition through up‐regulated immune checkpoint molecules (e.g., PD‐L1). Mechanistic investigations demonstrated that TIMD4 silencing substantially suppresses STAT3 and NF‐κB p65 phosphorylation, indicating TIMD4 may facilitate transcriptional activation of downstream pro‐proliferative and pro‐metastatic genes via dual regulation of STAT3/NF‐κB signalling axis crosstalk. These findings suggest TIMD4 targeting could not only directly inhibit tumour proliferation‐metastasis programs but also ameliorate immunosuppressive microenvironments through modulating immune checkpoint molecules like PD‐L1, thereby proposing potential combination strategies to counteract targeted therapy resistance [21]. To comprehensively assess the overall efficacy of ECGs, we established an EAS and further analysed its correlation with the immune microenvironment and immunotherapy outcomes. This study explored the potential impact of the EAS on patient stratification for immunotherapy by integrating multi‐omics data and immunotherapy cohorts. Pan‐cancer analysis demonstrated that patients with high EAS exhibited superior immunotherapy responses, a correlation independent of traditional biomarkers such as PD‐L1 expression and tumour mutation burden (TMB) [22]. Notably, a unique association between EAS and therapeutic resistance was observed in uterine corpus endometrial carcinoma (UCEC) and uveal melanoma (UVM), where the immunosuppressive microenvironment in UVM appeared to diminish the predictive utility of EAS. Based on these findings, we propose a clinical translation strategy: for most solid tumours, EAS could complement existing biomarkers by quantifying tumour‐specific features to optimise patient selection, whereas in specific cancer types (e.g., UCEC, UVM), microenvironmental characteristics (e.g., spatial immune cell distribution) should be incorporated into risk stratification to circumvent limitations of single‐marker approaches [23]. This strategy establishes a novel multidimensional screening framework for precision immunotherapy. In recent years, small molecule drugs have attracted significant attention from clinicians due to their excellent pharmacokinetic properties and strong targeting capabilities [24]. We found that there has been limited development of small molecule drugs targeting TIMD4. Therefore, based on structural biology methods, we performed molecular docking with FDA‐approved small molecules that showed positive correlations with TIMD4 expression and identified potential binding sites. This suggests that we have discovered a new strategy that can be combined with other anti‐tumour drugs.

The widespread use of immune checkpoint inhibitors in clinical practice has significantly changed the landscape of tumour treatment. However, the immunosuppressive tumour microenvironment greatly reduces the effectiveness of these immune checkpoint drugs [25]. As an integral influencing factor impacting the metastasis and treatment response of the cancer, the TME plays a significant role in cancer treatment and prognosis. Therefore, understanding the tumour microenvironment is crucial for discovering new effective targets for immunotherapy [26]. Epithelial–mesenchymal transition (EMT) is a critical process induced by TME signalling, which enhance the epithelial cells' invasiveness and promote metastasis and cancer progression [27]. It is evident that the ECGs significantly involved in the regulation of EMT in cancer. As one of the ECGs, TIMD4 is considerable to be a target for cancer treatment and prognosis. TIMD4 is a natural immune receptor that plays a key role in immune regulation. Recent data from tumour experimental models have shown that blocking TIMD4 can enhance the anti‐tumour efficacy of immune checkpoint inhibitors (ICI, anti‐PD1) and adoptive T‐cell (ACT) therapies in mouse models [28]. Antigen‐presenting cells (APCs) and cytotoxic T lymphocytes (CTLs) are important members of anti‐tumour immunity, which promote the tumour killing through cytotoxic granules and death receptor pathways [29]. And an impaired response of both leads to decreased immunotherapeutic response and tumour immune escape [30, 31]. TIMD4 on tumour‐associated macrophages (TAMs) can mediate the degradation of apoptotic tumour cells, thereby enhancing the reactivity of APCs and CTLs, ultimately weakening the efficacy of anti‐tumour chemotherapy [32]. In summary, TIMD4 plays an important regulatory role in the tumour immune microenvironment and may become a significant target for tumour immunotherapy and chemotherapy in the future.

Previous studies have indicated that the expression of TIMD4 is limited to antigen‐presenting cells; however, it has now been found to be expressed in various tumour cells. Recent research has shown that TIMD4 is up‐regulated in tissues of oesophageal cancer, colorectal cancer, pancreatic cancer, breast cancer and lung cancer, and it can lead to poor prognosis in lung cancer patients by promoting the growth and proliferation of NSCLC cells [33]. To explore the regulatory role of TIMD4 in tumour cells, we confirmed its expression and function in renal cell carcinoma. We found that TIMD4 expression was significantly higher in tumour cells than in normal cells, both at the protein and gene levels. Silencing TIMD4 effectively inhibited the proliferation and migration of tumour cells. As for in vivo experiments, we discovered that silencing TIMD4 could significantly suppress the growth of renal cell carcinoma. Overall, in tumour cells, TIMD4 acts as an oncogenic factor that promotes the progression of renal cell carcinoma and can serve as a novel anti‐cancer target to be used in combination with other anti‐cancer drugs to enhance their efficacy.

## 5 Conclusion

In this study, we utilised multi‐omics data to evaluate the expression differences, diagnostic and prognostic value of ECGs across various cancer types, and we also conducted preliminary explorations on some non‐tumour diseases. Our results indicate that ECGs are significantly correlated not only with tumour prognosis, but also with the tumour microenvironment, immunotherapy outcomes and chemotherapy sensitivity. We found that TIMD4 plays a role in antigen‐presenting cells and can also be expressed as a cancer‐promoting factor in tumour cells, affecting the prognosis of patients. Small molecule drugs targeting TIMD4, when combined with other anti‐tumour agents, hold promise as a new cancer treatment strategy. Finally, experimental validation demonstrated that silencing TIMD4 can inhibit the proliferation and invasion of renal cell carcinoma cells. Results of in vivo experiments showed that silencing TIMD4 inhibits the proliferation of renal cell carcinoma. In summary, our study comprehensively analysed the role and feasibility of ECGs as biomarkers for tumour diagnosis and prognosis using multi‐omics data, while also providing new biological targets for subsequent cancer treatment research. However, our study has certain limitations, such as not investigating the specific molecular mechanisms by which TIMD4 promotes renal cancer progression, which can be further validated through molecular biology experiments in the future.

## Author Contributions

**Hongze Li:** conceptualization (equal), formal analysis (equal), methodology (equal), project administration (equal), validation (equal), visualization (equal), writing – original draft (equal), writing – review and editing (equal). **Jiaqi Zhang:** data curation (equal), formal analysis (equal), methodology (equal), resources (equal), software (equal). **Yixiao Zhang:** conceptualization (equal), data curation (equal), validation (equal), visualization (equal), writing – original draft (equal), writing – review and editing (equal). **Zixuan Li:** software (supporting), supervision (supporting). **Yuxi Han:** conceptualization (equal), investigation (equal), methodology (equal), visualization (equal), writing – review and editing (equal). **Yu Lun:** conceptualization (equal), funding acquisition (lead), validation (equal), visualization (equal), writing – review and editing (equal).

## Ethics Statement

The authors have nothing to report.

## Consent

The authors have nothing to report.

## Conflicts of Interest

The authors declare no conflicts of interest.

## Supporting information


**Figure S1.** Mechanism validation and knockdown specific assays. (A) Downstream NF‐κB and STAT3 pathway activation after knockdown of TIMD4. (B) Effect of knockdown of TIMD4 expression using siRNA in 769‐P cell line. (C) Effect of knockdown of TIMD4 expression using siRNA in ACHN cell line.

**Appendix S1.** Supporting Information.

## Data Availability

The raw data of this study are freely available from the website TCGA Research Network (https://portal.gdc.cancer.gov/), GEO database(https://www.ncbi.nlm.nih.gov/geo/). Further inquiries can be directed to the corresponding authors.
