# Supplementary material for: A Pan‐Cancer Study of Tumour‐Associated Efferocytosis Core Genes and Preliminary Exploration of TIMD4 in Renal Cell Carcinoma
Source: J Cell Mol Med. 2025 Jun 27;29(12):e70671. doi: 10.1111/jcmm.70671 (PMC12203409; doi:10.1111/jcmm.70671)
Supplement: Supplementary file 1 — Figure S1. Mechanism validation and knockdown specific assays. (A) Downstream NF‐κB and STAT3 pathway activation after knockdown of TIMD4. (B) Effect of knockdown of TIMD4 expression using siRNA in 769‐P cell line. (C) Effect of knockdown of TIMD4 expression using siRNA in ACHN cell line. [file JCMM-29-e70671-s002.docx]

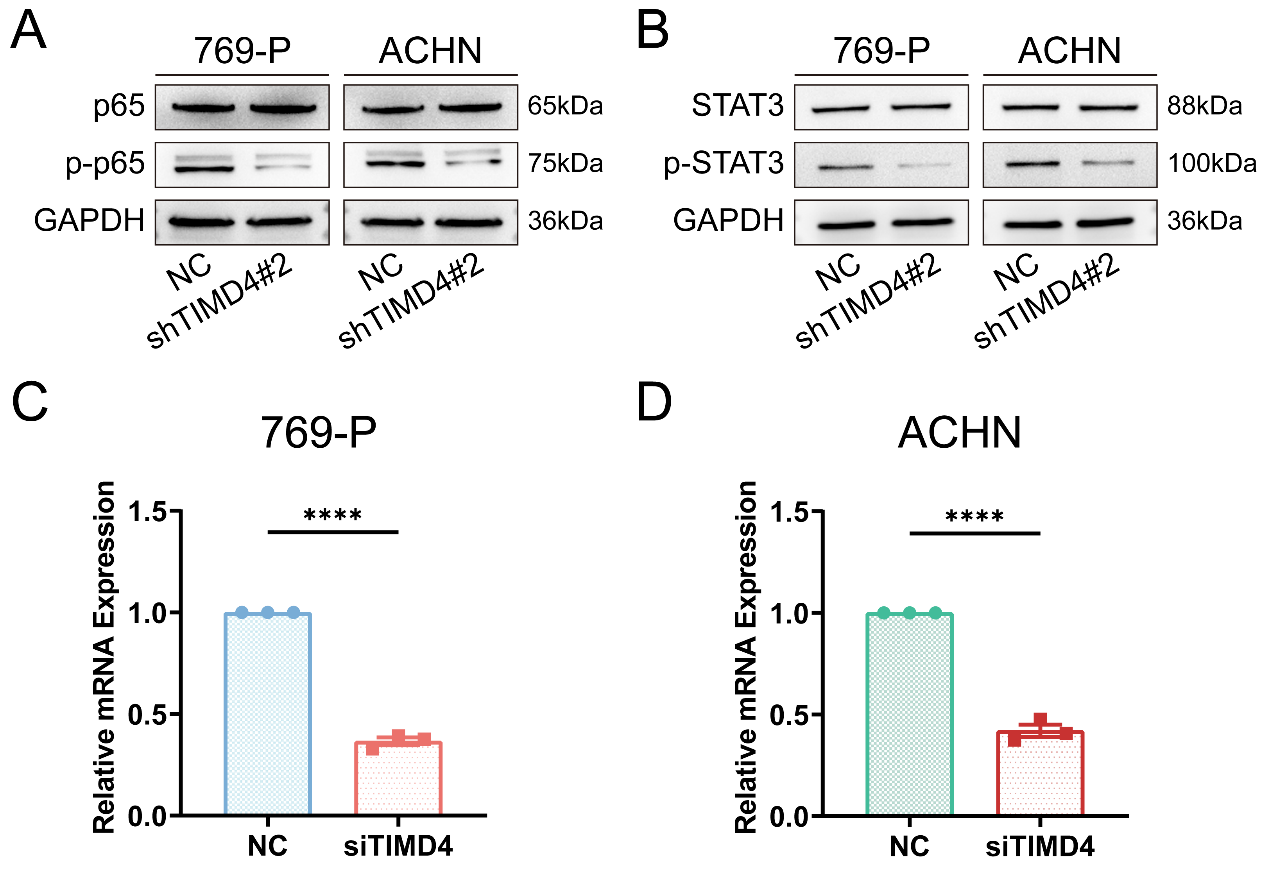


**Figure S1:** **Mechanism validation and knockdown specific assays.**

(A) Downstream NF-κB and STAT3 pathway activation after knockdown of TIMD4.

(B) Effect of knockdown of TIMD4 expression using siRNA in 769-P cell line.

(C) Effect of knockdown of TIMD4 expression using siRNA in ACHN cell line.
